# The “PharmFree” Campaign: Educating Medical Students about Industry Influence

**DOI:** 10.1371/journal.pmed.0030030

**Published:** 2006-01-31

**Authors:** Yavar Moghimi

## Abstract

Moghimi discusses an American Medical Students Association campaign that educates students about the influence of the pharmaceutical industry on medical training.

The American Medical Student Association (AMSA), with more than a half-century history of medical student activism, is the oldest and largest independent association of physicians-in-training in the United States. As the voice for over 50,000 members, AMSA has prided itself on fostering ideals such as honesty and integrity, and on promoting the interests of patients and communities.

In 2002, when AMSA launched its nationwide PharmFree campaign to educate medical students about the influence of the pharmaceutical industry on medical training ( http://www.amsa.org/prof/pharmfree.cfm), those ideals were being threatened by the lavish trips, gifts, and fancy meals that pharmaceutical companies were providing doctors to change their prescribing habits. With one pharmaceutical representative for every 15 practicing doctors [ 1], many doctors began to rely on representatives as a quick source of new information during their busy days. At the same time, most clinical programs lacked guidelines for how health-care professionals should deal with drug representatives [ 2]. Despite the lack of leadership on this issue from the mainstream medical community, idealistic medical students at AMSA rose to the occasion.

## The PharmFree Initiative

In 2002, the leaders of AMSA became concerned that the pharmaceutical industry's marketing tactics could compromise the personal and professional integrity of doctors. The PharmFree Initiative began as an educational campaign, in collaboration with No Free Lunch ( http://www.nofreelunch.org) and Healthy Skepticism ( http://healthyskepticism.org), encouraging all physicians-in-training and health-care providers to seek out evidence-based and unbiased sources of information, rather than relying on industry personnel for education.

To be a full member of the campaign, AMSA encouraged students to sign a pledge ( Box 1). Students who signed the pledge were given PharmFree paraphernalia, such as pens and badgeholders, to show their colleagues and patients that they were not being influenced by pharmaceutical company promotional tactics.

In response to the need for unbiased information on prescription drugs, AMSA forged ties with an independent, peer-reviewed nonprofit organization, called The Medical Letter, which offers critical evaluation of new drugs by authors with no ties to industry ( http://www.medletter.com). Members of AMSA were encouraged to use this source and other unbiased sources to learn about new drugs.

Even before the PharmFree campaign began, AMSA had already taken steps to ensure that their members were not exposed to pharmaceutical bias. Since 1978, AMSA banned pharmaceutical booths in the conference exhibit halls. Its monthly publication, *The New Physician*, is one of the few medical magazines free of pharmaceutical advertising ( http://www.amsa.org/tnp).

While there have been some notable campaigns by American medical organizations to advise their members of the pitfalls of accepting gifts, these organizations have themselves had close ties with industry, and AMSA wanted to avoid such ties. For example, in 2005, the American College of Physicians published its fifth edition of its *Ethics Manual* [ 3], which strongly discourages doctors from accepting “gifts, hospitality, trips and subsidies of all types” from industry. Yet the college's annual meeting is heavily sponsored by pharmaceutical companies, who pay $60,000 to sponsor the meeting's tote bags and $50,000 to sponsor the shuttle buses [ 4]. Similarly, when the American Medical Association mounted a $1 million campaign to educate its members about the ethics of accepting gifts from industry, most of the campaign's funding came from drug companies [ 5].

## The Impact of PharmFree

Since its inception, the PharmFree campaign has grown steadily and has gained recognition in the wider medical world. For example, the campaign has been cited in the *BMJ (British Medical Journal)* and the *Medical Journal of Australia* as a voice for change in the physician–drug company relationship [ 6, 7].

**Figure 1:**
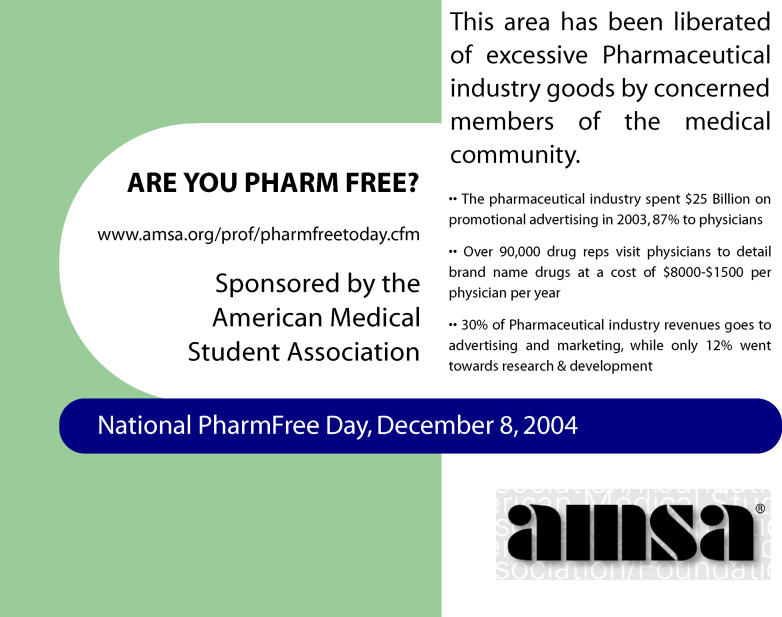
Promotional Material for AMSA's PharmFree Day

AMSA inaugurated PharmFree Day on December 8th of 2004. This was a day where local chapters across the country engaged in educational talks, recycled drug ads from medical journals and sent them back to the editors, exchanged pharmaceutical company pens for PharmFree pens, and “liberated” hospitals by putting PharmFree stickers ( Figure 1) over all the pharmaceutical logos that could be found (on pens, clocks, clipboards, etc). The day climaxed at the world headquarters of Pfizer, where students demonstrated and returned the pharmaceutical paraphernalia they had collected at their local chapters through a nationwide pen amnesty program ( http://www.amsa.org/news/focus/0305news.cfm). Pfizer's only response to these actions was calling the police, who allowed the protest to proceed because it was conducted on public property.

But there is still much work to be done to educate American medical students about the influence of industry marketing tactics. A recent national US survey of 1,143 third-year medical students, which had a response rate of 72.3%, found a mean exposure to pharmaceutical company gifts or sponsored events of one gift or event per week. It also found that most students believed they were entitled to gifts [ 8].

## The Future

The next step for the PharmFree campaign is the Counterdetailing Initiative, which will allow students to transform their passion for being PharmFree into grassroots activism. (“Detailing” is a term that describes the practice pharmaceutical companies use to educate doctors about their newest products through drug representatives.) AMSA members will be visiting doctor's offices distributing information sheets about unbiased sources of information, much like the way drug representatives “detail” doctors. Students who are on the wards can also participate by distributing pocket cards to their colleagues at drug company–sponsored events. In addition to encouraging our members to “just say no” to free lunch, AMSA is providing them with the tools to “counterdetail” drug representatives. Of course, our numbers pale in comparison to drug detailers, but “counterdetailing” is a symbolic gesture to show that we as medical students should be getting our information on new drugs from unbiased sources.

For more information on becoming a counterdetailer or on the PharmFree campaign, see http://www.amsa.org/prof/pharmfree.cfm. It is time for medical students around the world to revitalize the professionalism essential to being a doctor.

**Box 1. The AMSA PharmFree Pledge**

“I am committed to the practice of medicine in the best interests of patients and to the pursuit of education that is based on the best available evidence, rather than on advertising or promotion.

I, therefore, pledge to accept no money, gifts, or hospitality from the pharmaceutical industry; to seek unbiased sources of information and not rely on information disseminated by drug companies; and to avoid conflicts of interest in my medical education and practice.”

## Abbreviation

AMSA: American Medical Student Association
